# New Spin on an Old Fiber

**DOI:** 10.1289/ehp.112-a754

**Published:** 2004-09

**Authors:** Lance Frazer

Every year, cotton growers in the United States produce 20 million bales—some 9.6 billion pounds—of cotton fiber, or about one-fifth of total global production. The great majority of this fiber is destined for use in cloth, yet more than a quarter may never reach the fabric market: at each step throughout the production process, from harvesting the puffy white cotton bolls to weaving the cloth for the shirt you’re wearing as you read this, some portion of the fiber is lost to scrap or waste. Now a Cornell University researcher has developed a new process for electro-spinning waste cotton into nanofibers using a less harmful solvent, a change that could both profit the cotton industry and afford environmentally friendly applications.

According to Margaret Frey, an assistant professor of textile science at Cornell, some 4–8% of cotton fiber is lost at the textile mill in so-called opening and cleaning, which involves mechanically separating compressed clumps of fibers for removal of trapped debris. Another 1% is lost in drawing and roving—pulling lengths of fiber into longer and longer segments, which are then twisted together for strength. An average of 14–20% more is lost during combing and yarn production. Typically, waste cotton is used in relatively low-value products such as cotton balls, yarn, and cotton batting.

Cotton is 90% cellulose—a very pure source of this fiber. Perhaps, Frey theorizes, more productive use could be made of this waste cotton. “My idea,” she says, “was to . . . give the industry a way to produce some high-end products.”

Frey’s process involves dissolving the cotton with ethylene diamine, a relatively benign solvent, and using an electrospinning process to produce fibers 100 times smaller than anything obtainable by conventional spinning technologies. In electrospinning, a polymer solution is pulled by an arcing electrical charge through the air and onto an electrical ground. Electrospun materials can then be incorporated into a traditionally woven product to add strength or durability.

Frey says the great thing about nanofibers is that they have a very high surface-to-volume ratio, so much less material will accomplish more. For example, she says, adding no more than 0.1 gram of nanofiber material per square meter to conventional filter material—for example, in a biohazard suit or air filter—will dramatically improve the efficiency of the filter.

“The military can also use it in protective systems for soldiers at risk from chemical or biological weapons,” Frey says. “The tremendous filtration capabilities can protect personnel without making them feel like they’re wrapped in plastic.” Frey also suggests that these fibers could be made into mats that could absorb fertilizers, pesticides, and similar substances, later releasing them in a timed, targeted fashion.

## Fiber Options

Frey says cellulose has a large number of hydrogen bond sites. “The trick, then,” she says, “is to break these hydrogen bonds without depolymerizing the cellulose and turning it back into its basic constituent—glucose. Once we have a good solvent for cellulose, we’ll be able to process it into any shape we want: small fibers, films, and so on.” At the same time, another challenge is to find a solvent that is benign, yet volatile enough to work with electrospinning. “In electro-spinning, you apply a voltage to a solution,” Frey explains. “As that charge arcs across to a ground, the solvent needs to evaporate across the path of the arc so that what you collect is fiber.” Frey thinks ethylene diamine may be just the solvent to meet both these needs.

The solvents typically used to convert cellulose into a soluble compound—for example, to process wood pulp into rayon—include carbon disulfide and sodium hydroxide, both of which carry substantial health baggage. Carbon disulfide, in the impure form typically used in industrial processes, has an odor that has been likened to rotting radishes. Breathing low concentrations of carbon disulfide over extended periods can cause headache, tiredness, and nerve damage, while breathing high concentrations can be fatal. The vapor also combusts and explodes easily. Sodium hydroxide is a room-temperature solid that generates tremendous heat when dissolved in water or neutralized with acid, and can react with a variety of metals to create flammable hydrogen gas. It can cause irritation of the skin and eyes at low exposure, while higher concentrations can cause severe burns to the eyes, skin, and gastrointestinal tract. Frey says rayon production in the United States resulted in a number of Superfund sites.

In the early 1990s, a second cellulose fiber, Lyocell, became available, created using amine oxides as the solvent. Amine oxides dissolve cellulose without the issues of the chemical reactions in rayon manufacturing, says Frey, but it’s not as flexible a process as the rayon process—the types of fibers that can be made have properties in a fairly limited range.

“Amine oxides . . . have a number of very reactive nitrogen–oxygen bonds,” explains Richard Kotek, an assistant professor in North Carolina State University’s Department of Textile Engineering, Chemistry, and Science. He adds, “Those oxides can not only have a lot of side reactions, they can also decompose very rapidly if the temperature is too high.”

Frey believes her system, using ethylene diamine, offers the best of both worlds. “It’s a direct solvent system, with no unwanted side reactions,” she says, “where we can capture and reuse the solvent and use the process to produce fibers for a full range of end users.”

## Cottoning to a New Technology

Frey developed the process in collaboration with assistant professor Yong Joo and graduate student Choowon Kim, both from Cornell’s School of Chemical and Biomolecular Engineering. The team discovered that ethylene diamine swells cellulose and separates individual polymer chains from each other without dissolving the molecules.

To actually dissolve the cellulose, they added thiocyanate salts. In experiments presented at the fall 2003 American Chemical Society national meeting, dried cellulose was dispersed in ethylene diamine using a vortex mixer. Sodium thiocyanate or potassium thiocyanate was added, and the mix was blended again.

The samples were then put through a freeze-and-thaw system to complete the dissolution. Samples were frozen at –20°C for four hours, then thawed in a 40°C water bath for 20 minutes. Three cycles of freezing and thawing were sufficient to complete dissolution in all samples where dissolution occurred (in samples where solutions didn’t form, no number of cycle repetitions was sufficient to trigger solution formation; the reason why this is so is one subject of ongoing research). “We’re not sure why [the freeze-and-thaw system] works,” says Frey, “but we have several thermodynamic studies ongoing to find out.” The solutions were then successfully electrospun.

Discovery of ethylene diamine as the solvent of choice for Frey’s process was based partly on previous research and partly on serendipity. In recent years, an ammonia-based solvent with a high vapor pressure had been produced and shown to be effective with cellulose. However, the vapor pressure was “too high for what we wanted,” says Frey. “So we went up to the next large molecule, which is hydrazine [a colorless liquid used in rocket fuels and chemical manufacturing, which can cause nervous system, kidney, and liver damage]. Hydrazine’s issues are pretty well documented, so we went up to the next larger molecule, which was ethylene diamine.” She says one advantage to ethylene diamine is that it remains a liquid at room temperature, unlike ammonia-based solvents, which volatilize at or near room temperature.

## Hurdles to High Cotton

Frey admits there have been some perceptual hurdles to overcome with regards to providing another use for waste cotton. “There is this concern that if you’re dealing with a waste product, you somehow have an ‘impure’ stream, and that has caused a distraction [within the industry],” she says. “But I think it’s a distraction of perception, rather than reality.”

Don Bailey, vice president of textile research and implementation for the industry research organization Cotton Incorporated, is cautiously interested in Frey’s process, with some reservations. Bailey has worked with textiles science since 1971. “In that time,” he says, “every solvent I’ve ever run across that was supposed to be the next best solvent for textile processing has been banned or heavily restricted shortly thereafter. That’s why I don’t generally get too excited when I hear people talk about solvents.”

Bailey also points to the question of basic economics. “The original rayons used cotton waste fibers, but the industry found they just weren’t able to compete economically with wood pulp,” he says. “And even though there’s a cotton oversupply in the world today, it would take some serious economic studies to see if [Frey’s] process was competitive.”

Finally, he says, is the question of whether and how the process might alter the cotton fibers. As an example, he points to bamboo fibers. “There’s a lot of bamboo in the world, so people think it will make a great feedstock for fiber production and might offer unique properties,” he says. “But one problem is that while the bamboo itself is mildew-resistant, the processed fiber loses those properties. Problematic changes like this require more study.”

The environmental friendliness of the process is also still somewhat debatable. “Solvents do, for the most part, have attendant health and environmental issues,” Frey says. “We still have concerns with [ethylene diamine], as we do with most solvents. The Environmental Protection Agency has not identified it as a problem, and there are no specific regulations covering its usage, but it still needs to be recaptured and recycled, like any other solvent.”

It should also be noted that the thiocyanates added to make the process work have their own risks, including respiratory irritation, weakness, low blood pressure, and death at doses of 15–30 grams. And if heated to decomposition, they can release toxic fumes of ammonia, nitrogen oxides, and cyanide.

Ultimately, says Bailey, if the new process proves itself to be environmentally benign, “that would be great, but I’ll need to see a long-term proven track record first. . . . If it can indeed make nanofibers through electrospinning, it would be of interest to industry.”

“I think [Frey’s] approach is an excellent idea,” says Kotek. “Nanofibers are very popular, and although I don’t see a multitude of applications for nanofibers in industry at this time, it’s certainly something that could find a number of applications in the near future.”

Frey agrees. “So far, there are no limitations to the types of cellulose that can be dissolved using the process we’ve developed,” she says. “We have tried a wide range of samples, including cotton, wood pulps, bacterial cellulose, and anything else I could get my hands on. I think the process has tremendous potential.”

## Figures and Tables

**Figure:**
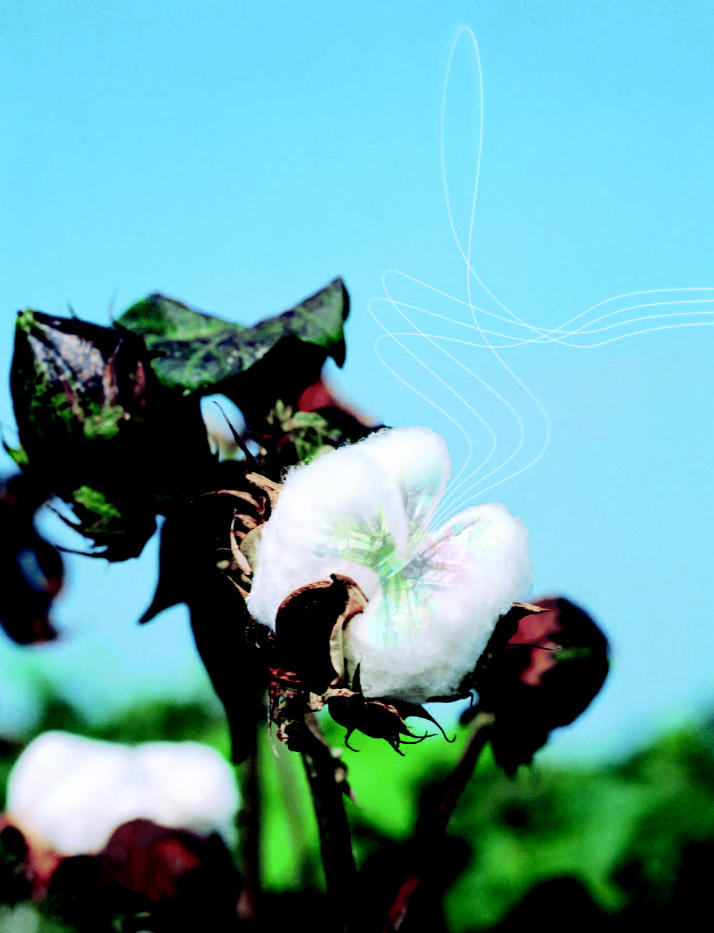


**Figure:**
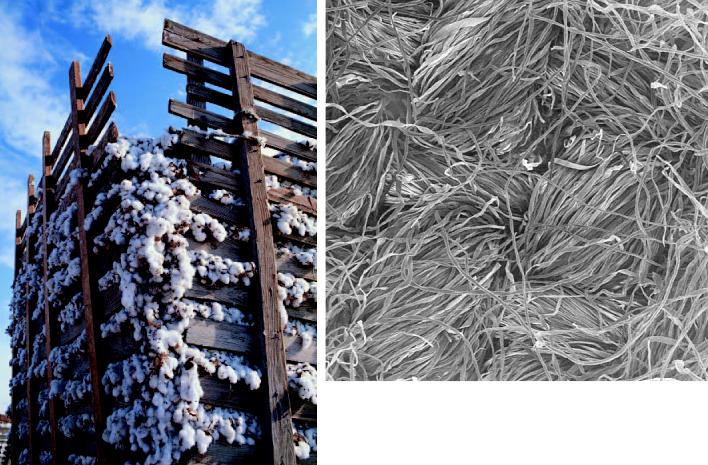
**Spinning gold out of waste?** Nearly a quarter of the 9.6 billion pounds of cotton fiber produced each year in the United States is lost to waste during harvesting, transport, and processing. A new technology uses electrospinning of waste cotton fibers, made possible by addition of a relatively benign solvent, to create high-value nanofibers (above).
